# Using Cancer-Associated Fibroblasts as a Shear-Wave Elastography Imaging Biomarker to Predict Anti-PD-1 Efficacy of Triple-Negative Breast Cancer

**DOI:** 10.3390/ijms26083525

**Published:** 2025-04-09

**Authors:** Zhiming Zhang, Shuyu Liang, Dongdong Zheng, Shiyu Wang, Jin Zhou, Ziqi Wang, Yunxia Huang, Cai Chang, Yuanyuan Wang, Yi Guo, Shichong Zhou

**Affiliations:** 1 Department of Ultrasonography, Fudan University Shanghai Cancer Center, Shanghai 200032, China; 2 Department of Oncology, Shanghai Medical College, Fudan University, Shanghai 200032, China; 3 School of Information Science and Technology, Fudan University, Shanghai 200433, China

**Keywords:** TNBC, shear-wave elastography, deep-learning, cancer-associated fibroblast, immune checkpoint inhibition

## Abstract

In the clinical setting, the efficacy of single-agent immune checkpoint inhibitors (ICIs) in triple-negative breast cancer (TNBC) remains suboptimal. Therefore, there is a pressing need to develop predictive biomarkers to identify non-responders. Considering that cancer-associated fibroblasts (CAFs) represent an integral component of the tumor microenvironment that affects the stiffness of solid tumors on shear-wave elastography (SWE) imaging, wound healing CAFs (WH CAFs) were identified in highly heterogeneous TNBC. This subtype highly expressed vitronectin (VTN) and constituted the majority of CAFs. Moreover, WH CAFs were negatively correlated with CD8^+^ T cell infiltration levels and influenced tumor proliferation in the Eo771 mouse model. Furthermore, multi-omics analysis validated its role in immunosuppression. In order to non-invasively classify patients as responders or non-responders to ICI monotherapy, a deep learning model was constructed to classify the level of WH CAFs based on SWE imaging. As anticipated, this model effectively distinguished the level of WH CAFs in tumors. Based on the classification of the level of WH CAFs, while tumors with a high level of WH CAFs were found to exhibit a poor response to anti programmed cell death protein 1 (PD-1) monotherapy, they were responsive to the combination of anti-PD-1 and erdafitinib, a selective fibroblast growth factor receptor (FGFR) inhibitor. Overall, these findings establish a reference for a novel non-invasive method for predicting ICI efficacy to guide the selection of TNBC patients for precision treatment in clinical settings.

## 1. Introduction

With advances in immunotherapy, immune checkpoint inhibitors (ICIs) have demonstrated potential in the treatment of triple-negative breast cancer (TNBC) [1,2]. However, the response rate of ICI monotherapy for the treatment of TNBC in clinical practice remains abysmal, and the treatment is associated with severe immunotherapy-related adverse events [3,4,5]. For these non-responsive patients, combination immunotherapy is typically considered. Therefore, there is an urgent need to develop predictive biomarkers for identifying non-responders in the clinical setting.

At present, existing biomarkers used to predict immunotherapy response rely on invasive methods such as programmed cell death protein 1 (PD-1) and programmed cell death ligand 1 (PD-L1) expression [6,7]. TNBC, however, is a highly heterogeneous disease [8]. Consequently, the heterogeneous distribution of tumors and unique expression patterns during cancer progression can result in inaccurate evaluation and diagnosis [9,10]. Recently, radiomics has emerged as an innovative method to explore the relationship between imaging features of tumors and their gene expression to predict immunotherapy response [11,12]. However, due to the substantial gap between phenotypes in molecular biology, this method has lacked biological interpretation. Thus, an improved and more interpretable biomarker is warranted to more accurately predict treatment response.

Triple-negative breast cancer is characterized by high levels of fibrosis and an increase in collagen fiber deposition driven by cancer-associated fibroblasts (CAFs) [13,14]. As a key member of the tumor microenvironment (TME), CAFs play an essential role in the immunosuppressive microenvironment and may serve as biomarkers for predicting the efficacy of immunotherapy [15,16,17]. The stiffness of solid tumors is caused by the activation of fibroblasts into CAFs, which produce excessive extracellular matrices [18]. Shear-wave elastography (SWE) imaging, an advanced ultrasound imaging technique, has emerged as a new tool to visualize and quantify the stiffness of solid tumors. Compared with magnetic resonance imaging (MRI) or positron emission tomography (PET) -based radiomics, SWE offers real-time, non-invasive, and low-cost assessment of tumor stiffness, indirectly reflecting the immune microenvironment [19,20]. Given the high degree of fibrosis in TNBC, the proportion of CAFs in tumors directly affects SWE imaging features. Hence, SWE is a potential tool for reflecting and quantifying CAFs. Nevertheless, CAFs exhibit a high degree of heterogeneity. Consequently, some subtypes may not be directly associated with tumor stiffness [21,22]. Our previous studies established that wound healing CAFs (WH CAFs, vitronectin^high^) were the predominant CAF subtype under physiological states and largely determined tumor stiffness [23].

**Scheme 1:**
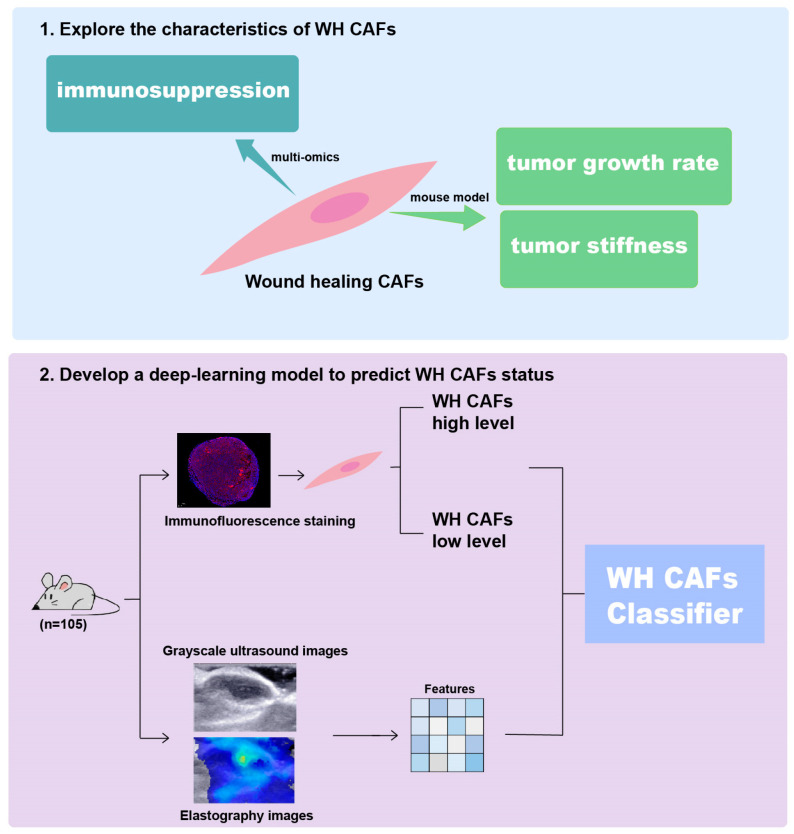

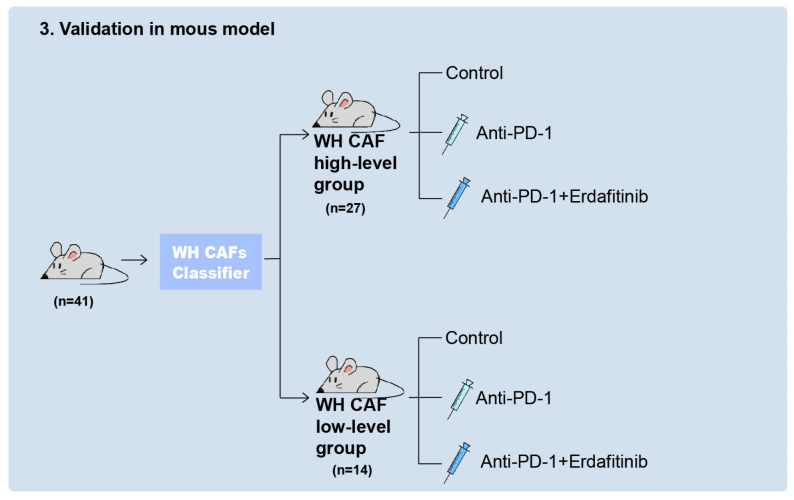
Schematic illustration of this study outline. Firstly, we explored the characteristics of wound healing CAFs (WH CAFs). We found that WH CAFs were correlated with immunosuppression through multi-omics, and had an impact on tumor growth rate and stiffness in a mouse model. Then, we developed a deep-learning model to predict the status of WH CAFs based on shear-wave elastography (SWE) imaging and immunofluorescence (IF) staining. Finally, we verified the immunosuppressive effect of WH CAFs and the efficiency of this model.

In this study, we characterized the features of WH CAFs and confirmed their role in immune suppression. Based on SWE images, a deep-learning model was trained to distinguish the proportion of WH CAFs and classify tumors as WH CAF high-level group and low-level group. As expected, tumors with high levels of WH CAFs had a poor response to anti-PD-1 monotherapy and responded to the combination of anti-PD-1 and erdafitinib, a selective fibroblast growth factor receptor (FGFR) inhibitor. Overall, our study confirmed the feasibility of predicting the efficacy of ICIs in TNBC through a deep learning model based on SWE imaging, and is expected to guide the precise treatment of patients with ICIs in a non-invasive manner in the future (Scheme 1).

## 2. Results

### 2.1. WH CAFs Are Correlated with Immunosuppression

In our previous study [23], WH CAFs were identified as a major component of TNBC CAFs and a key determinant of extracellular matrix (ECM) stiffness. Considering the significance of CAFs in TNBC immunosuppression, WH CAFs in immunoregulation were further characterized.

**Figure 1:**
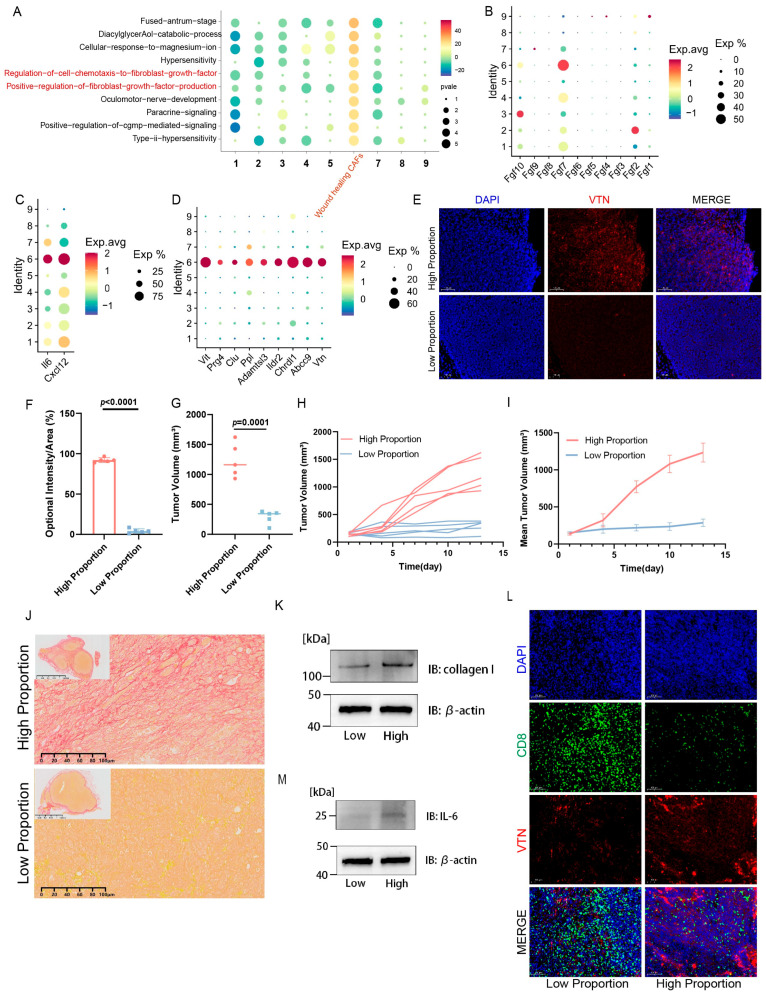
Wound-healing CAFs are correlated with immunosuppression and tumor growth rate. (**A**) Gene ontology biological process (GOBP) enrichment analysis was performed based on CAF gene expression in different CAF subtypes and the focused pathways and subtypes are marked in red. (**B**) Dot plot showing different types of fibroblast growth factor (FGF) expression in various subtypes of CAFs. (**C**) Interleukin-6 (IL-6) and C-X-C motif chemokine ligand 12 (CXCL12) expression in each subtype of CAFs. (**D**) Several proteins that are only highly expressed on WH CAFs. (**E**) Immunofluorescence staining of vitronectin (VTN) (red) distribution in tumors, scale bar: 100 μm. (**F**) VTN immunofluorescence quantification results of selected tumors (n = 5 independent samples). (**G**) Comparison of tumor volume from high- and low-proportion groups on the last day (n = 5 independent samples). (**H**) Selected tumor volume curves from high- and low-proportion groups over 14 days (n = 5 independent samples). (**I**) Mean selected tumor volume curve comparison from high- and low-proportion groups over 14 days (n = 5 independent samples). (**J**) Sirius red staining in high and low groups, scale bar: 1.25 mm (low magnification), 100 μm (high magnification). (**K**,**M**) Western blot analysis of collagen I and IL-6 protein in high- and low-proportion groups. (**L**) The immunofluorescence co-staining image of CD8 (green) and VTN (red), scale bar: 100 μm (1: Extracellular Matrix CAFs; 2: Matrix CAFs 1; 3: Development CAFs; 4: Antigen presentation CAFs 1; 5: Antigen presentation CAFs 2; 6: Wound healing CAFs; 7: Vascular CAFs; 8: Inflammatory CAFs; 9: Matrix CAFs 2). Data expressed as mean ± S.E.M; statistical comparisons were performed using Student’s *t* test.

Previously published single-cell sequencing (sc-RNA seq) data were analyzed, revealing that WH CAFs were a subgroup that positively regulated fibroblast growth factor (FGF) (Figure 1A). The FGF/FGFR signaling axis can promote various diseases, especially malignancies [24]. Of note, across all subclusters, WH CAFs (cluster 6) had high levels of FGF7, which interacts with FGFR2 (Figure 1B). FGFR2 is a membrane-spanning tyrosine kinase that mediates signaling from FGFs [25], and its activation of FGFR2 drives tumorigenesis and immune evasion [26,27,28].

Given the established link between CAFs and immunosuppression [15,16,17], WH CAFs were observed to express immune-suppressive factors such as interleukin-6 (IL-6) and C-X-C motif chemokine ligand 12 (CXCL12) highly (Figure 1C). Additionally, CAFs have been reported to regulate T-cells, mediating immune response and suppression in the TME via secretion of IL-6/17/10 and CXCL12 [21]. These findings collectively indicate that WH CAFs play a key role in the immune suppression of TNBC.

In the present study, several markers of WH CAFs were characterized (Figure 1D). Among them, vitronectin (VTN) is a protein-coding gene encoding vitronectin, which is an adhesive glycoprotein promoting cell adhesion in ECM through fibroblast activation and proliferation [29,30]. In addition, earlier studies noted a high score of ten CAF-associated genes, including VTN, related to immune evasion and poorer ICI efficacy [31]. Given the involvement of WH CAFs in ECM remodeling and TME modulation, VTN was selected as a marker for WH CAFs. The aforementioned results collectively suggest that WH CAFs might be related to immune suppression in tumors.

In order to further investigate the role of WH CAFs in immunosuppression in vivo, forty Eo771 mouse models were developed. After performing immunofluorescence (IF) staining on tumors and quantifying VTN levels, a significant variation in VTN levels was noted among mice, indicating significant variability in the content of WH CAFs (Figure 1E). Next, we selected sections for immunofluorescence assessment at one-third intervals along the long axis of the tumor to represent the WH CAF proportion of the tumor and then calculated the proportion of VTN fluorescence relative to the entire tumor. Ten tumors with the highest and lowest proportion of WH CAFs were selected (Figure 1F), and their tumor growth rates were examined. The results showed that the tumor growth rate in the group with a high proportion of WH CAFs was significantly higher than that in the group with a low proportion (Figure 1G–I).

As is well documented, tissue fibrosis and ECM stiffening promote tumor progression [32]. Thus, Sirius staining was performed, revealing that the tumors in the high group had higher fibrosis levels than those in the low group (Figure 1J). Furthermore, Western blot analysis exposed that the expression level of collagen I was higher in tumors in the high WH CAF group compared to the low WH CAF group (Figure 1K).

IF assays were performed to examine the co-expression of CD8 and VTN to investigate the relationship between immune cells and WH CAFs in the tumor microenvironment (Figure 1L). It turned out that in the high expression group of VTN, the expression of CD8 was lower, while in the low expression group of VTN, the expression of CD8 was higher. We quantified the fluorescence results of VTN and CD8 and tested the correlation between them. Our results and analysis using Spearman’s correlation coefficient (i.e., Spearman’s r) revealed a linear, inverse correlation between the expression of VTN and CD8 (Supplementary Figure S1). Notably, the results of Western blot analysis demonstrated that the expression level of IL-6 was much higher in the high-proportion group, consistent with the sc-RNA seq data (Figure 1M).

Overall, these results indicated that WH CAFs, as a subtype of CAFs highly expressing FGF, might be associated with tumor stiffness and immunosuppression.

### 2.2. Multi-Omics Further Confirm That Tumors with a High Proportion of WH CAFs Exhibit Greater Immune Suppression

Multi-omics assessment plays a vital role in the discovery of tumor markers. In order to further assess the predictive utility of WH CAFs for the efficacy of immunotherapy in TNBC, RNA-seq, metabolism, and protein mass spectrum data were compared in the 10 tumors with the highest and lowest proportions of WH CAFs.

**Figure 2:**
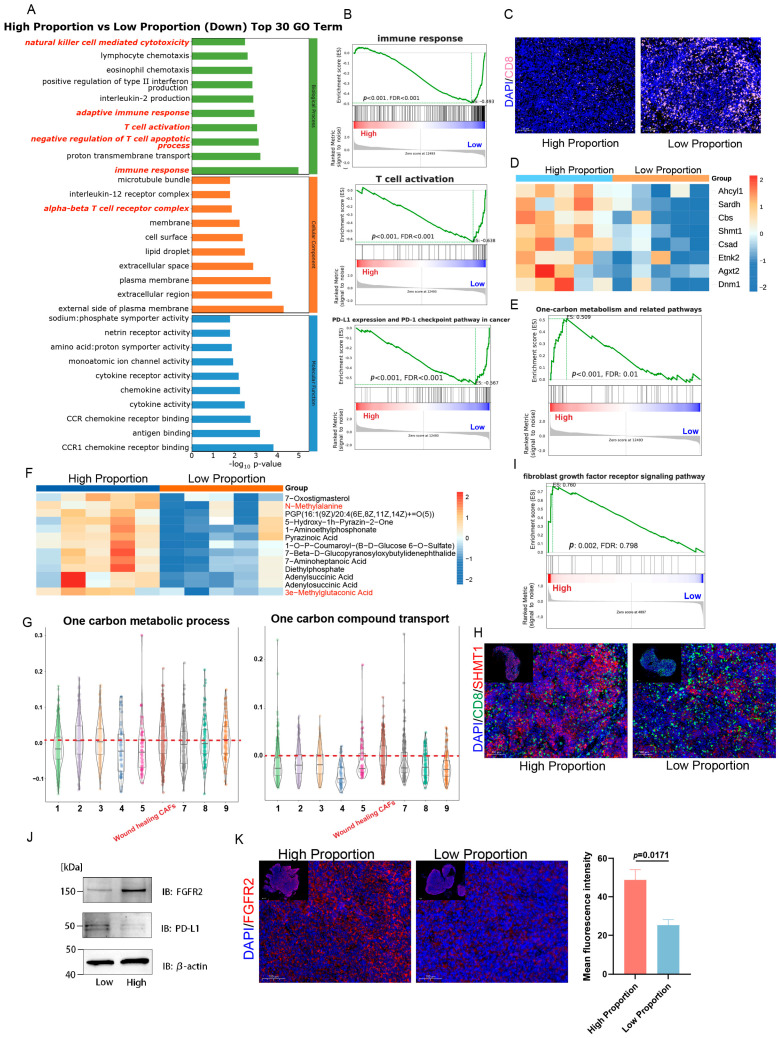
Woundhealing CAF high-proportion tumors exhibit greater immune suppression. (**A**) Gene ontology (GO) enrichment analysis revealed the downregulation of the top 30 pathways between high- and low-proportion groups. Focus pathways were marked in red. (**B**) Gene set enrichment analysis (GSEA) of immune response, T cell activation, programmed cell death protein 1 (PD-L1) expression, and PD-1 checkpoint pathway in cancer between high- and low-proportion groups. (**C**) Immunofluorescence staining of CD8 (pink) expression distribution in tumor tissues, scale bar: 100 μm. (**D**,**E**) Heatmap and GSEA showed one-carbon metabolism and related pathways in high- and low-proportion groups. (**F**) Various forms of metabolism in high- and low-proportion groups. Methylation was marked in red. (**G**) Addmodulescore in pathway (one carbon metabolic process and one carbon compound transport) based on gene expression of different CAF subtypes, focus subtypes marked in red, and the red dashed line represents the median value of WH CAFs as the reference line. (**H**) The immunofluorescence co-staining image of serine hydroxymethyltransferase (SHMT) (red) and CD8 (green), scale bar: 2000 μm (low magnification), 100 μm (high magnification). (**I**) GSEA of FGFR signaling pathway in high and low groups. (**J**) FGFR2 and PD-L1 protein expression in high- and low-proportion groups. (**K**) Immunofluorescence staining of fibroblast growth factor receptor2 (FGFR2) (red) between high- and low-proportion groups, and quantitative results, scale bar: 2000 μm (low magnification), 100 μm (high magnification). (n = 5 independent samples) (1: Extracellular Matrix CAFs; 2: Matrix CAFs 1; 3: Development CAFs; 4: Antigen presentation CAFs 1; 5: Antigen presentation CAFs 2; 6: Wound healing CAFs; 7: Vascular CAFs; 8: Inflammatory CAFs; 9: Matrix CAFs 2). Data expressed as mean ± S.E.M; statistical comparisons were performed using Student’s *t* test.

Based on differentially expressed genes, gene ontology (GO) enrichment analysis revealed the downregulation of immune-related pathways in tumors with a high proportion of WH CAFs (Figure 2A). At the same time, gene set enrichment analysis (GSEA) was carried out, revealing the inhibition of pathways related to the immune response, such as T cell activation, PD-L1 expression, and PD-1 checkpoint in cancer (Figure 2B, Supplementary Figure S2A). Taken together, these results indicate that tumors with a high proportion of WH CAFs displayed a poorer immune response. IF staining demonstrated that the WH CAF high-proportion group had lower T cell infiltration levels compared to the WH CAFs low-proportion group (Figure 2C).

GSEA and differential metabolomics data demonstrated that the expression of one-carbon metabolism and related pathways in the high-proportion group was higher compared to the low-proportion group (Figure 2D,E, Supplementary Figure S2B). One-carbon metabolism is known to facilitate cancer cell proliferation and growth by providing precursors for nucleotides and amino acids, which are hallmarks of metabolic reprogramming [33,34]. According to a study, various forms of methylation via enhancing one-carbon metabolism regulate malignancy in cancer cells [35]. In addition, our metabolomics data indicated significant differences in methylation levels between the two groups (Figure 2F). Consistently, we also found in our single-cell sequencing data mentioned before that the one-carbon unit-related pathways in WH CAFs were higher than those in other CAF subtypes (Figure 2G). Serine hydroxymethyltransferase (SHMT) catalyzes a key reaction in one-carbon metabolism [36]. Immunofluorescence co-localization staining of CD8 and SHMT1 showed that in the low-proportion group with high CD8 expression, SHMT1 expression was low, while in the high-proportion group with low CD8 expression, SHMT1 expression was high (Figure 2H). These results conjointly suggest that tumors with a high proportion of WH CAFs might exhibit elevated activity in one-carbon metabolism, potentially accounting for their higher tumor growth rate.

Importantly, the FGFR signaling pathway was up-regulated in the high-proportion group (Figure 2I). In addition, the expression levels of the FGFR-related proteins Cep57, Ll17rd, and Fgfbp1, were significantly up-regulated (Supplementary Figure S2C). Following the extraction of protein from tumors, the expression of FGFR2 and PD-L1 was detected via Western blot, and the results were in line with those of the above-mentioned sequencing data (Figure 2J). Moreover, immunofluorescence staining was conducted to validate the expression of FGFR2 between the two groups (Figure 2K). These results were in agreement with the sc-RNA seq data, implying that WH CAFs are a subgroup that positively regulates FGF.

In summary, these results demonstrated the suppression of immune-related pathways in tumors with a high proportion of WH CAFs, whilst sequencing data exposed that WH CAFs were characterized by the up-regulation of one-carbon metabolism and the FGFR signaling pathway.

### 2.3. Deep Learning Model Predicts WH CAF Level from SWE Imaging

Considering the association between WH CAFs and immune suppression, we hypothesized that the high proportion of WH CAFs may potentially impact the tumor’s response to immunotherapy. Therefore, to non-invasively distinguish whether the tumor is in a WH CAF high proportion situation or not, a deep learning model was generated based on SWE imaging.

**Figure 3:**
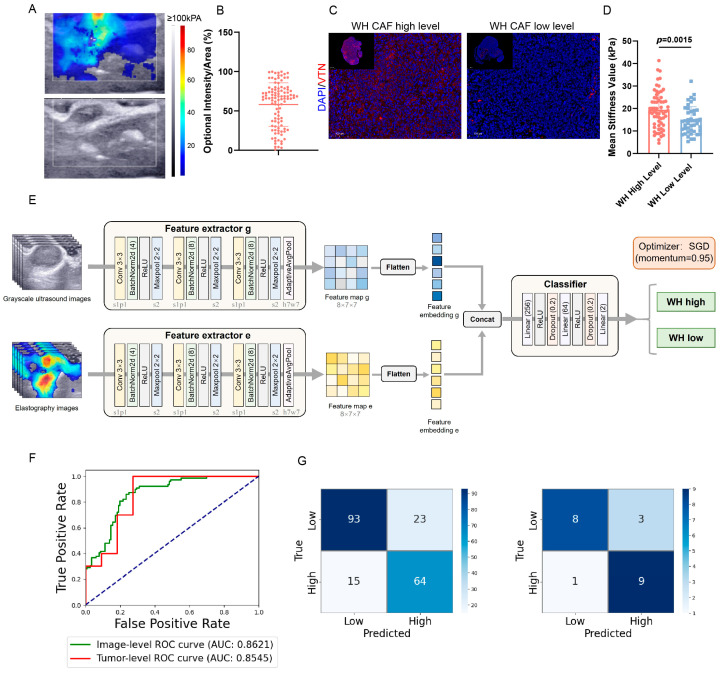
Deep learning model to evaluate WH CAF level. (**A**) Representative SWE image of mice; each image is composed of a pseudo-color image and grayscale image. (**B**) Quantification results of VTN immunofluorescence staining. The solid red line represents the average (n = 105 independent samples). (**C**) Representative immunofluorescence staining of VTN (red) from high and low level, scale bar: 100 μm. (**D**) Mean stiffness value in high- and low-level groups (WH high-level group: n = 62, WH low-level group: n = 43). (**E**) The framework of our proposed deep learning model. (**F**) Performance of the deep learning model to predict the WH CAFs level. ROC: receiver operating characteristic curve; AUC, area under the curves. (**G**) Confusion matrices in two classification categories. The confusion matrices showed the pairwise comparison. Data expressed as mean ± S.E.M; statistical comparisons were performed using Student’s *t* test.

Afterward, another batch of Eo771 mouse model was constructed, and two weeks after tumor formation, SWE was performed (Figure 3A). Then, the expression of VTN was detected within the tumor. We selected sections for immunofluorescence assessment at one-third intervals along the long axis of the tumors to represent the WH CAF proportion of the tumors. Interestingly, a significant difference was observed in the quantification results of VTN in tumors from different mice (Figure 3B). Subsequently, the average of these 105 quantification results was calculated to establish a threshold to divide tumors into two groups, namely the WH CAF high-level and WH CAF low-level groups (Figure 3C). Comparing the mean stiffness of tumors revealed that the stiffness in the high group was higher than that of the low group (Figure 3D). Overall, a high level of WH CAFs was associated with higher stiffness.

Given that WH CAFs in tumors could be non-invasively visualized by SWE imaging, a deep learning model was trained to classify WH CAF levels using bimodal image pairs (Figure 3E). The model extracted and integrated image features from grayscale ultrasound images and elastography images to yield the final prediction. For the WH CAF level classification task, a total of 105 tumors with paired bimodal images were employed. Noteworthily, each tumor generally contained several bimodal image pairs. The model was trained to initially perform image-level classifications, making predictions for each image pair corresponding to each tumor. For tumor-level prediction, the proposed model initially generated image-level predictions and subsequently applied a voting mechanism to determine the prediction for each tumor. Our proposed model achieved an area under the curve (AUC), accuracy, sensitivity, and specificity of 86.21%, 80.51%, 81.01%, and 80.17% at the image level, and 85.45%, 80.95%, 90.00%, and 72.73% at the tumor level, respectively (Figure 3F, Table 1). Moreover, our model also achieved strong performance in cross-validation (Supplementary Table S1), confirming its effectiveness and reliability. The confusion matrix further highlighted the satisfactory performance of the pioneered model in both classification categories (Figure 3G).

Additionally, ablation studies were performed to compare the performance of our proposed bimodal model with unimodal models that used either grayscale ultrasound images or elastography images alone. Ablation studies at both the image level and tumor level demonstrated that our bimodal model outperformed the unimodal models (Table 2 and Table 3), highlighting the effectiveness of bimodal fusion. To enhance the interpretability of the model, we employed Grad-CAM visualization to intuitively demonstrate which regions of the SWE images contributed the most to the classification. The results revealed that the tumor regions, especially in areas with higher stiffness values, contributed the most to the model’s classification (Supplementary Figure S3).

Overall, a deep learning model that could predict high or low levels of WH CAFs was established. However, further validation was warranted to confirm the impact of WH CAFs on the efficacy of immunotherapy.

### 2.4. FGFR Inhibitor Enhances Therapeutic Responses to ICIs in Tumors by Inhibiting WH CAFs

Previous studies have described that patients with low levels of PD-L1 and tumor-infiltrating lymphocytes (TIL) may derive more benefits from combination therapy, which was designed to enhance T cell infiltration into tumors while concomitantly preventing T cell inactivation [37]. Erdafitinib, a selective FGFR inhibitor, has been reported to reverse T cell exclusion in immune-excluded tumor types by blocking FGFR signaling from CAFs, thereby enhancing the effect of anti-PD-1 [26]. Considering that WH CAFs are a subtype closely related to FGFR, the combination of erdafitinib and anti-PD-1 was analyzed.

**Figure 4:**
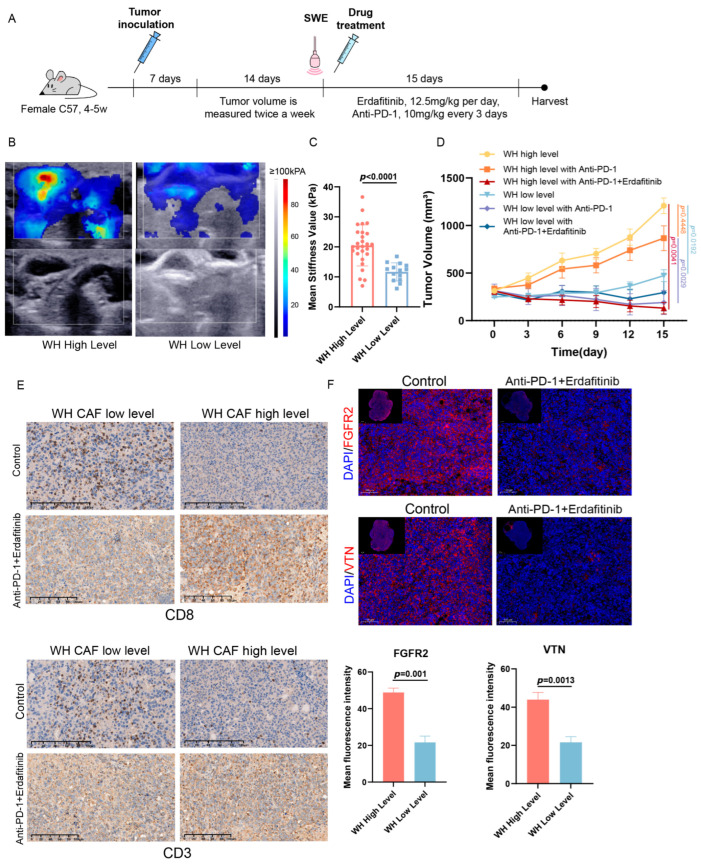
FGFR inhibitor potentiates immune checkpoint inhibitors (ICIs) in WH CAF high-level tumors. (**A**) Schematic of the experimental protocol. SWE: shear wave elastography. (**B**) Representative SWE images of tumors in high-level and low-level groups at the end of volume measurement. (**C**) Mean stiffness value of tumors in high-level and low-level groups (WH high-level group: n = 27, WH low-level group: n = 14). (**D**) Tumor volume curves in different groups during the 15-day monitoring period (Group: WH CAFs high level, WH CAFs high level with Anti-PD-1, WH CAFs high level with Anti-PD-1+Erdafitinib: n = 9; group: WH CAFs low level, WH CAFs low level with Anti-PD-1: n = 5; group: WH CAFs low level with Anti-PD-1+Erdafitinib: n = 4). (**E**) Comparison of immunohistochemical results of CD8 and CD3 in high- and low-level groups before and after Anti-PD-1+Erdafitinib treatment, scale: 100 μm. (**F**) Immunofluorescence staining and quantization results of FGFR2 and VTN proteins in WH CAFs high-level group before and after Anti-PD-1+Erdafitinib treatment, scale bar: 2000 μm (low magnification), 100 μm (high magnification). Data expressed as Mean ± S.E.M; statistical comparisons were performed using Student’s *t* test.

To confirm our hypothesis that tumors with high levels of WH CAFs were less responsive to anti- PD-1 monotherapy, the Eo771 mouse model was constructed. Then, tumor volume was recorded on the seventh day following tumor implantation. In order to mitigate the influence of tumor size on drug efficacy, tumors of similar volume were selected for SWE imaging and subsequent drug treatments (Figure 4A). The image features collected were then incorporated into the deep learning model, yielding classification results regarding the high and low levels of WH CAFs. Among the 41 mice, 27 were assigned to the WH CAF high-level group, while the remaining 14 were assigned to the WH CAF low-level group. Figure 4B illustrates representative SWE images. The measurement results revealed that the high group had a higher stiffness compared to the low group (Figure 4C), consistent with the above-mentioned results.

Next, mice were randomly divided into six groups (WH CAF high-level (as control), WH CAF high-level with Anti-PD-1, WH CAF high-level with Anti-PD-1+Erdafitinib; WH CAF low-level (as control), WH CAF low-level with Anti-PD-1, and WH CAF low-level with Anti-PD-1+Erdafitinib). Drug treatments were administered over 21 days (Figure 4A). As anticipated, the tumor growth rates in the WH CAF high-level groups were higher than those in the WH CAF low-level groups (*p* = 0.0192, η^2^ = 0.4375). Likewise, the effect of PD-1 monotherapy was significantly higher in the low-level groups compared to the high-level groups (*p* = 0.0029, η^2^ = 0.6056). In the WH CAF high-level with anti-PD-1 group, the effect of PD-1 monotherapy was comparable to that in the WH CAF high-level group (*p* = 0.4448, η^2^ = 0.05951); in contrast, tumor volume control was significant in the combination with erdafitinib group (*p* = 0.0041, η^2^ = 0.5772) (Figure 4D).

Figure 4E displays the protein expression levels of CD3 and CD8 in tumors. In tumors of the WH CAF high-level group, the level of CD8^+^ T cell infiltration was lower compared to the WH CAF low-level group. However, after combination treatment, the degree of CD8^+^ T cell infiltration was comparable between both groups. The expression levels of VTN and FGFR2 in the WH CAF high-level group decreased after combination treatment (Figure 4F), indicating that erdafitinib enhanced the effect of anti-PD-1 by targeting the FGFR2-related pathway as well as WH CAFs.

Overall, these results validated that the developed deep-learning model could classify WH CAF levels in tumors. It is worthwhile emphasizing that tumors with high levels of WH CAFs were not responsive to anti-PD-1 monotherapy but responsive to the combination of anti-PD-1 therapy and FGFR inhibitor. Indeed, our findings demonstrated that WH CAFs could indeed serve as a new biomarker for identifying tumors responding to either anti-PD-1 monotherapy or combination treatment.

## 3. Discussion

Herein, a novel approach was proposed to predict the efficacy of anti-PD-1 therapy in TNBC by identifying WH CAFs as a new biomarker. Our results revealed that tumors with a high proportion of WH CAFs were immune-suppressed. Thus, we posited a potential relationship between WH CAFs and ICI efficacy. Then, the role of WH CAFs in immunosuppression was assessed through animal models and multi-omics analyses. Next, a noninvasive and comprehensive method to assess the level of WH CAFs was developed. Considering that WH CAFs were the subtype with the highest proportion under physiological conditions and largely determined tumor stiffness [23], SWE was utilized to visualize WH CAFs in solid tumors. Based on SWE imaging and immunofluorescence staining, a deep-learning model classifier was developed to classify the level of WH CAFs and it was observed that the response of tumors to ICIs significantly varied with different levels of WH CAFs. Overall, this study validated that WH CAFs could serve as a biomarker in TNBC for predicting the response of tumors to ICIs or combination treatment. In other words, a new method was developed to predict the effect of ICIs in TNBC through SWE imaging. The clinical relevance of our findings lies in the potential to use SWE imaging as a non-invasive tool for predicting treatment response, thereby guiding the selection of TNBC patients for precision treatment and providing non-pathological imaging support for the formulation of clinical immunotherapy protocols. Furthermore, our discovery that tumors with high levels of WH CAFs are less responsive to anti-PD-1 monotherapy but benefit from combination treatment with the FGFR inhibitor erdafitinib suggests a personalized approach to immunotherapy that could optimize patient outcomes and minimize the risk of adverse effects.

At present, the diagnosis and prediction of diseases rely heavily on biopsies. As an emerging tool, deep learning radiomics of SWE offers several advantages over traditional needle biopsies [19,20]. Ascribed to the high heterogeneity of TNBC, local biopsies may not accurately reflect the overall condition of the tumor [9,10]. However, SWE can capture images from multiple angles and perform repeated acquisitions, providing a more comprehensive view of the tumor’s internal status. Moreover, SWE is non-invasive, repeatable, and real-time, making it ideal for monitoring tumor progression and providing rapid feedback on therapeutic efficacy while avoiding the side effects of multiple punctures. Meanwhile, using deep learning, researchers have significantly improved the precision of disease diagnosis, treatment, and prognosis prediction of breast cancer by extracting a large number of features from medical images and combining them with advanced algorithms [38,39].

Although numerous studies have explored radiomics for non-invasively predicting the efficacy of immunotherapy [11,12], these studies were primarily based on traditional immune biomarkers, such as PD-L1, T-cell infiltration, tumor mutational burden, etc., and aimed to predict immunotherapy efficacy by establishing relationships between imaging characteristics and gene expression. However, tumorigenesis is an intricate process, with multiple gaps in our understanding of the interactions between molecules, phenotypes, and imaging characteristics, which limits the biological interpretability of these approaches. In addition, although studies have demonstrated a correlation between SWE parameters and the pathological characteristics of breast cancer [40], the direct use of SWE images for tumor diagnosis or prediction remains controversial due to their limited and unstable precision [41,42,43,44]. Therefore, there is a need for a biomarker that is linked to SWE images and can reflect the tumor microenvironment, serving as a link between SWE imaging and tumor biology.

Notably, numerous studies have explored the regulatory role of CAFs in TME [15,16,37]. CAFs are highly heterogeneous cells, signifying that different CAF subtypes are responsible for distinct functions [45,46]. Bartoschek et al. [47] classified CAFs into four subtypes: vascular CAFs, matrix CAFs, cycling CAFs, and developmental CAFs. Grauel et al. [48] utilized single-cell RNA sequencing and spatial transcriptomics to classify CAFs in the 4T1 mouse breast cancer model into four subtypes: inflammatory CAFs, myofibroblastic CAFs, vascular CAFs, and proliferative CAFs. These classifications highlighted the functional diversity of CAFs and their roles in shaping the tumor microenvironment and influencing tumor progression. In our previous research, we divided CAFs into nine subtypes, and WH CAFs, which are relevant to stiffness and immunosuppression, were further investigated herein. Indeed, targeting specific subtypes can further achieve clinical precision in diagnosis and treatment. This study connected the characteristics of SWE imaging with WH CAFs by exploring tumor stiffness, thus establishing a link between imaging features and cellular and molecular aspects which enhanced the interpretability of imaging predictions.

Nevertheless, some limitations of our study cannot be overlooked. To begin, the sample size of the mouse models used in the study was limited and external validation was not performed, which may have compromised the generalizability of the findings. The predictive accuracy and applicability of the deep learning model in clinical settings warrant further validation in larger and more diverse patient populations. Future research should focus on validating the predictive value of WH CAFs in larger human cohorts and diverse patient populations. Additionally, the mechanism of WH CAFs promoting the tumor immunosuppressive microenvironment needs to be further explored. We found that WH CAFs were a subgroup which had high levels of IL-6 and CXCL12, and numerous studies have revealed the mechanisms by which IL-6 and CXCL12 promote immunosuppression in tumors as immunosuppressive factors [49,50,51,52,53,54,55]. Meanwhile, WH CAFs increased tumor stiffness, which primarily affected the immune microenvironment through influencing the function and abundance of T cells in tumors, as a physical barrier [56,57,58,59]. Hence, WH CAFs may promote an immunosuppressive tumor microenvironment through mechanisms potentially related to the regulation of immune factors and tumor stiffness, but further investigation is needed in the future. The exploration of the molecular mechanisms underlying the interaction between WH CAFs and the immune response, as well as the role of the FGFR signaling pathway in mediating this interaction, could provide further insights into the development of combination therapies.

## 4. Materials and Methods

### 4.1. Cell Lines

Eo771 cells were cultured in DMEM medium supplemented with 10% FBS and 1% Penicillin-Streptomycin. The cells were incubated in an incubator at 37 °C with 5% CO_2_.

### 4.2. Mice and Treatments

C57BL/6JGpt (4 weeks old, female) were purchased from GemPharmatech (Nanjing, China). All the animal experiments were performed according to the protocols approved by the department of laboratory animal science, Fudan University.

Then, 5 × 10^4^ E0771 cells in PBS were mixed with matrigel with a 1:1 ratio (*v*/*v*). The cells were resuspended in 100 μL and injected subcutaneously into the mammary fat pad of 4-week-old C57BL/6JGpt female mice. At 7 days after tumor inoculation, the tumor volume was measured twice a week, and the volume was calculated using the standard modified formula $volume=\frac{{width}^{2}\times length}{2}$. When the tumor size reached 250 mm^3^, drug treatment was initiated. FGFR inhibitor Erdafitinib (Selleck, Houston, TX, USA, #JNJ-42756493) was administered by oral gavage once every other day at 12.5 mg/kg, and Anti-PD-1 antibody (Bioxcell, Lebanon, NH, USA, #857122D1) was injected intraperitoneally every 3 days at 10 mg/kg for 15 days.

### 4.3. Immunofluorescence (IF)

Immunofluorescence was performed on Formalin-fixed paraffin-embedded (FFPE) sections from a mouse model. The primary antibodies used for IF staining include VTN antibody (Proteintech, Wuhan, China, 66398-1-lg), FGFR2 antibody (Proteintech, Wuhan, China, 13042-1-AP), CD8a antibody (Proteintech, Wuhan, China, 29896-1-AP), and SHMT1 antibody (Proteintech, Wuhan, China, 67963-1-Ig).

All steps were conducted by skilled laboratory personnel for the preparation of tissue sections and IF staining. These individuals were unaware of the tissue sample groupings throughout the procedure, focusing solely on the technical execution of the tasks without involvement in the subsequent data analysis.

### 4.4. Immunohistochemistry (IHC)

IHC was performed on Formalin-fixed paraffin-embedded sections from a mouse model. The primary antibodies used for IHC include CD3a antibody (Proteintech, Wuhan, China, 17617-1-AP) and CD8a antibody (Proteintech, Wuhan, China, 29896-1-AP).

All steps were conducted by skilled laboratory personnel for the preparation of tissue sections and IHC staining. These individuals were unaware of the tissue sample groupings throughout the procedure, focusing solely on the technical execution of the tasks without involvement in the subsequent data analysis.

### 4.5. Western Blot

Tumor tissues were dissected into fragments and transferred to 1.5 mL Eppendorf (EP) tubes along with steel grinding balls. The tissues were homogenized with 500 μL of RIPA lysis buffer supplemented with phenylmethylsulfonyl fluoride (PMSF). The samples were then processed using a tissue lyser (Shanghai Jing Xin, Shanghai, China, JXFSTPRP-CL) at 4 °C for 30 min with a frequency of 30 cycles per 30 s. The resulting supernatant was collected, and protein concentrations were determined using a BCA protein assay kit (Thermo Fisher Scientific, Waltham, MA, USA, catalog number 23227). Protein lysates were combined with loading buffer, and the mixtures were heated to 95–100 °C to facilitate protein denaturation.

For electrophoresis, the protein samples were loaded onto an SDS-polyacrylamide gel, and the resolved proteins were subsequently blotted onto a polyvinylidene fluoride (PVDF) membrane. The membrane was then saturated with a blocking solution to mask non-specific binding sites. It was incubated with the primary antibody at 4 °C for an extended period, followed by a series of washes with Tris-buffered saline and Tween 20 (TBST). Afterward, the membrane was incubated with a horseradish peroxidase (HRP)-conjugated secondary antibody (Proteintech, Wuhan, China, SA00001-2) at ambient temperature for approximately one hour.

Primary antibodies include FGFR2 antibody (ABclonal, Wuhan, China, A19051), PD-L1 antibody (Proteintech, Wuhan, China, 82719-15-RR), IL-6 antibody (Proteintech, Wuhan, China, 66146-1-Ig), collagen I antibody (CST, Danvers, MA, USA, #91144), and β-actin (Proteintech, Wuhan, China, 66009-1-Ig).

### 4.6. SWE Imaging of Tumors

SWE imaging was performed on the mouse tumors before the drug treatment. All SWE imaging operations were performed by the same ultrasound physician with clinical experience using the same equipment. The equipment was a Supersonic Aixplorer ultrasound system using a linear array probe (7–15 MHz). The probe was positioned perpendicular to the long axis of the tumor for imaging. The settings used were gain: 48%, range: 100 kPa, S5/0: 75%, B mode: 49. On the color map provided by the machine, blue indicated soft tissue and red indicated hard tissue. Then, the image was frozen and the tissue elasticity was measured using the Q-BOX and recorded as kPa. The region of interest (ROI) was the entire tumor area. SWE imaging was performed several times for each tumor (10 times on average). Each elastography image has its corresponding grayscale image.

### 4.7. Development of the Deep Learning Model

We designed a bimodal deep learning model to perform WH CAF level classification based on paired grayscale ultrasound images and elastography images of the tumor ROI region (Figure 3E). Given the small sample size in this study, we selected the mean value of quantified results from 105 tumors as the threshold for WH CAFs level stratification. We utilized two feature extractors, ‘Feature extractor g’ and ‘Feature extractor e’, to encode images from two different modalities into feature maps. The two feature extractors shared the same structure. All convolutional layers used a kernel size of 3 × 3, with a stride of 1 and padding of 1. The number of channels for each BatchNorm2d layer was indicated in brackets next to the layer name. All MaxPool layers used a kernel size of 2 × 2, and the AdaptiveAvgPool layer was configured with a height and width of 7 × 7. These feature maps were then flattened, concatenated, and fed into the ‘Classifier’, which produced the final prediction of WH CAF level. In the classifier, the output feature sizes of the Linear layers were 256, 64, and 2, respectively. The dropout layers were set with a probability of 0.2. The activation functions used throughout the model were ReLU, and their locations were depicted in the architecture figure. The optimizer was the SGD optimizer with the momentum set to 0.95.

We trained the model using cross entropy loss, which is defined as

$$L=-\left(ylog\left(p\right)+\left(1-y\right)\log\left(1-p\right)\right)$$

where y is the true label and p is the predicted probability of the model.

### 4.8. Experiment Setting of the Deep Learning Model

To obtain the WH CAF classification label for deep learning training, we sorted the WH CAF quantification results and set the median value as the threshold; tumors with quantification values exceeding the threshold were labeled as ‘WH high’, and those with quantification values below the threshold were labeled as ‘WH low’. All the tumors were divided into training set, validation set, and test set according to the ratio of 3:1:1. SWE imaging was performed several times for each tumor, resulting in several ‘grayscale image–elastography image’ pairs (10 times on average). The number of tumors and bimodal image pairs in each data set are shown in Table 4. Training set and validation set were used to train the model and select best model parameters, and the test set was used to evaluate the model’s performance.

All images were resized to 224 × 224 before being input to the model. We performed data augmentation in the training set using random horizontal and vertical flips. The learning rate and batch size were set using a grid search algorithm (Supplementary Figure S4), with the hyperparameter values achieving the highest sum of AUC, accuracy, sensitivity, and specificity at the image level in the validation set being selected. We experimented with a learning rate of ${1\times 10}^{-2},{1\times 10}^{-3},{1\times 10}^{-4},{1\times 10}^{-5},{1\times 10}^{-6}$ and batch size of 2, 4, 8, 16, 32, 64, 128. The model performed best at a learning rate of 1 × 10^−4^ and a batch size of 32, which were ultimately used in this study. The number of training epochs was set as 200. The proposed deep learning model was implemented on the Pytorch platform and trained using an NVIDIA GTX 3090.

### 4.9. Evaluation Metrics for WH CAF Level Classification

We utilized four metrics to evaluate the deep learning model’s performance, including AUC, accuracy, sensitivity, and specificity. AUC quantifies the model’s ability to distinguish between positive and negative classes by calculating the area under the Receiver Operating Characteristic curve, which plots the true positive rate against the false positive rate at different threshold levels. Accuracy is the ratio of the number of correctly predicted samples to the total number of samples, which is defined as

$$Accuracy=\frac{TP+TN}{TP+TN+FP+FN}$$

where TP is true positives, TN is true negatives, FP is false positives, and FN is false negatives. Sensitivity measures the proportion of actual positives that are correctly identified by the model, which is defined as

$$Sensitivity=\frac{TP}{TP+FN}$$

And specificity measures the proportion of actual negatives that are correctly identified by the model, defined as

$$Specificity=\frac{TN}{TN+FP}$$

Higher AUC, accuracy, sensitivity, and specificity indicate better classification performance.

### 4.10. RNA Sequencing and Data Analysis

Eo771 tumor samples were collected and then total RNA was purified using Trizol. RNA purity and quantification were evaluated using the NanoDrop 2000 spectrophotometer (Thermo Scientific, Waltham, MA, USA). RNA integrity was assessed using the Agilent 2100 Bioanalyzer (Agilent Technologies, Santa Clara, CA, USA). Then, the libraries were constructed using VAHTS Universal V6 RNA-seq Library Prep Kit. The subsequent transcriptome sequencing and data analysis were executed by OE Biotech Co., Ltd., located in Shanghai, China.

The analysis of differential expression was conducted employing the DESeq2 algorithm. *p* value < 0.05 and foldchange > 2 or foldchange < 0.5 was set as the threshold for significantly differential expression genes (DEGs).

### 4.11. 4D-DIA Quantitative Proteomics and Data Analysis

Eo771 tumor samples were collected. Then, liquid nitrogen was added and they were ground thoroughly. An appropriate amount of the sample was transferred into a 1.5 mL centrifuge tube, and sample lysis buffer, phosphatase inhibitors, and the protease inhibitor PMSF added to a final concentration of 1 mM. A cold homogenizer was used to grind the sample at −35 °C, 60 Hz, for 120 s, and then this process was repeated once more. After grinding was complete, the solution was centrifuged at 4 °C and 12,000 rpm for 10 min and the supernatant collected. The supernatant was the total protein solution of the sample, and the protein concentration was determined using the BCA method. The subsequent transcriptome sequencing and data analysis were executed by OE Biotech Co., Ltd., located in Shanghai, China.

Raw DIA data were processed using Spectronaut Pulsar™ version 18.3 (Biognosys, Schlieren, Switzerland) software. Proteins were considered significantly upregulated when *p* value < 0.05 and foldchange > 2, and significantly downregulated when *p* value < 0.05 and foldchange < 0.5.

### 4.12. Metabolomic and Data Analysis

Eo771 tumor samples were collected. First, 30 mg of the sample was weighed into a 1.5 mL centrifuge tube, then two small steel balls and 600 μL of methanol–water (*v*/*v* = 4:1, containing mixed internal standards, 4 μg/mL) was added; it was placed in a −40 °C freezer for 2 min, then ground in a mill (60 Hz, 2 min); the extract was sonicated in an ice water bath for 10 min and left it to stand overnight at −40 °C; centrifuged at low temperature for 10 min (12,000 rpm, 4 °C); and a syringe was used to draw up 150 μL of the supernatant, which was then filtered through a 0.22 μm organic phase syringe filter before being transferred to an LC sample vial and stored at −80 °C until LC-MS analysis. Simultaneously, 150 μL of the supernatant was placed into a glass derivatization vial and the sample was dried down using a centrifugal concentrator; 80 μL of methoxyamine hydrochloride pyridine solution (15 mg/mL) was added to the glass derivatization vial, and it was incubated in a 37 °C shaking incubator for 60 min to carry out the oxime reaction; after taking out the sample, 50 μL of BSTFA derivatization reagent and 20 μL of n-hexane were added, along with 10 μL of 10 internal standards (C8/C9/C10/C12/C14/C16/C18/C20/C22/C24, all prepared in chloroform), and reacted at 70 °C for 60 min; and after taking out the sample, it was left to stand at room temperature for 30 min before proceeding with GC-MS metabolomics analysis. The subsequent transcriptome sequencing and data analysis were executed by OE Biotech Co., Ltd., located in Shanghai, China.

Differential metabolites were selected with VIP values greater than 1.0 and *p*-values less than 0.05.

### 4.13. Single-Cell RNA Sequences Analysis

In the single-cell data analysis, we utilized published research data from our team [23]. The control group (n = 2) received no treatment; the PD-1 group was treated with PD-1 monoclonal antibody (n = 2) at a dosage of 10 mg/kg, administered every two days; and the PD-1 combined with nanoparticle group (n = 2) was treated with nanoparticles carrying artesunate, delivered via PLGA as the carrier, with a calculated artesunate dosage of 30 mg/kg, administered every three days. On the seventh day following the inoculation of cells in the mouse mammary fat pad, the tumors were collected for single-cell sequencing after a continuous monitoring period of 18 days. The data analysis focused on the expression of WH CAFs across all three groups.

### 4.14. Differential Gene Expression and Pathway Enrichment

*p* value < 0.05 and foldchange > 2 or foldchange < 0.5 were set as the threshold for significantly differential expression genes. Utilizing the hypergeometric distribution, we conducted enrichment analyses for differentially expressed genes across GO, KEGG, Reactome, and WikiPathways pathways.

### 4.15. GSEA

GSEA was performed using GSEA software (https://cloud.oebiotech.cn/spa#/bio/detail?number=ec6f9bd8-d104-4c35-ba1f-3f85b3192669 (accessed on 1 April 2025)). It was performed on all the genes detected in high-proportion and low-proportion groups to test whether the specific gene set was enriched at the top or bottom of the ranking list.

### 4.16. Statistical Analysis

Prism 9.0 software (GraphPad Software, Boston, MA, USA) was used for statistical analysis. The data were collected from at least two independent experiments and all data in different experimental groups were used and expressed as mean ± S.E.M. Differences between groups were tested with a *t*-test. The significance of differences is indicated at * *p* < 0.05, ** *p* < 0.01, *** *p* < 0.001, and **** *p* < 0.0001.

## 5. Conclusions

In summary, our study offers a new perspective on the role of CAFs in TNBC and highlights the potential of SWE imaging in predicting treatment response to immunotherapy. These findings highlight the importance of considering the tumor microenvironment in clinical decision-making, and are expected to guide the precise treatment of patients with ICIs in a non-invasive manner in the future.

## Figures and Tables

**Table 1:** The metrics of our proposed deep learning model in WH CAF level prediction.

	AUC (%)	Accuracy (%)	Sensitivity (%)	Specificity (%)
Image-level	86.21	80.51	81.01	80.17
Tumor-level	85.45	80.95	90.00	72.73

**Table 2:** The results of ablation study at the image level.

Image-Level	AUC (%)	Accuracy (%)	Sensitivity (%)	Specificity (%)
Unimodal-g	82.09	66.15	98.73	43.97
Unimodal-e	79.12	66.67	97.47	45.69
Bimodal-g and e	86.21	80.51	81.01	80.17

**Table 3:** The results of ablation study at the tumor level.

Tumor-Level	AUC (%)	Accuracy (%)	Sensitivity (%)	Specificity (%)
Unimodal-g	83.64	71.43	100.0	45.45
Unimodal-e	78.18	61.90	100.0	27.27
Bimodal-g and e	85.45	80.95	90.00	72.73

**Table 4:** The number of tumors and bimodal image pairs in training, validation, and test sets.

	Training	Validation	Test
Tumors	63	21	21
Bimodal image pairs	600	207	195

## Data Availability

The datasets used and analyzed during the study are available from the corresponding author on reasonable request.

## Supplementary Materials

The supporting information can be downloaded at: https://www.mdpi.com/article/10.3390/ijms26083525/s1.

## Abbreviations

TNBC	triple-negative breast cancer
ICIs	immune checkpoint inhibitors
CAFs	cancer-associated fibroblasts
WH CAFs	wound-healing CAFs
VTN	vitronectin
PD-1	programmed cell death protein 1
PD-L1	programmed cell death ligand 1
FGFR	fibroblast growth factor receptor
TME	tumor microenvironment
SWE	shear-wave elastography
MRI	magnetic resonance imaging
PET	positron emission tomography
ECM	extracellular matrix
sc-RNA seq	single-cell sequencing
IL-6	interleukin-6
CXCL12	C-X-C motif chemokine ligand 12
IF	immunofluorescence staining
GO	gene ontology
GOBP	gene ontology biological process
GSEA	gene set enrichment analysis
TIL	tumor-infiltrating lymphocytes
SHMT	serine hydroxymethyltransferase
AUC	area under the curve
ROC	receiver operating characteristic curve
DEGs	differential expression genes
