# Supplementary material for: Using Cancer-Associated Fibroblasts as a Shear-Wave Elastography Imaging Biomarker to Predict Anti-PD-1 Efficacy of Triple-Negative Breast Cancer
Source: Int J Mol Sci. 2025 Apr 9;26(8):3525. doi: 10.3390/ijms26083525 (PMC12027048; doi:10.3390/ijms26083525)
Supplement: Supplementary file 1 [file ijms-26-03525-s001.zip › supplementary figures S1-S4 table S1.pdf]

Using Cancer-Associated Fibroblasts as a Shear-Wave Elastography Imaging Biomarker to Predict Anti-PD-1 Efficacy of Triple-Negative Breast Cancer

Zhiming Zhang [1,2,†], Shuyu Liang [3,†], Dongdong Zheng [1,2], Shiyu Wang [1,2], Jin Zhou [1,2], Ziqi Wang [1,2], Yunxia Huang [1,2], Cai Chang [1,2], Yuanyuan Wang [3], Yi Guo [3,*] and Shichong Zhou [1,2,*]

[1] Department of Ultrasonography, Fudan University Shanghai Cancer Center, Shanghai 200032, China
[2] Department of Oncology, Shanghai Medical College, Fudan University, Shanghai 200032, China
[3] School of Information Science and Technology, Fudan University, Shanghai 200433, China
* Correspondence: guoyi@fudan.edu.cn (Y.G.); sczhou@hotmail.com or sczhou@fudan.edu.cn (S.Z.)
† These authors contributed equally to this work.

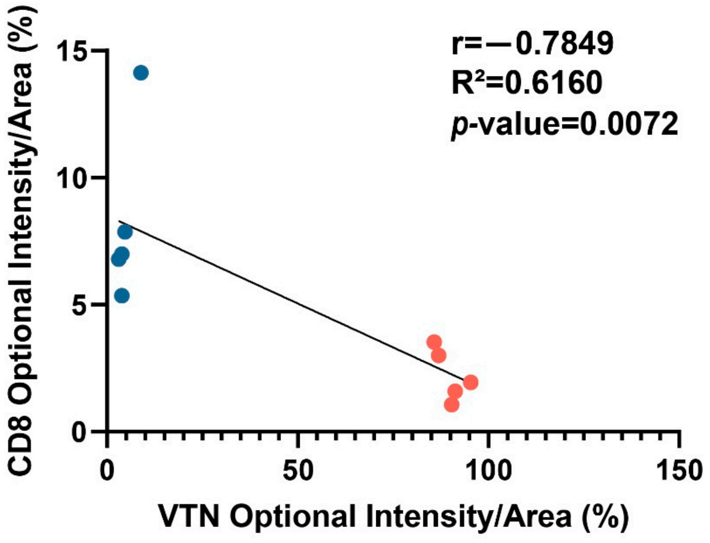

Supplementary Figure S1. Correlation analysis of the quantification results from immunofluorescence staining of CD8 and VTN. The scatter plot shows the distribution of the quantified results for VTN and CD8. Red dots represent tumors with a high proportion of WH CAFs, while blue dots represent tumors with a low proportion of WH CAFs. The Spearman's r and p-value and the R^2value of the best linear fit to the experimental data are shown to quantify the strength of the correlations (n=5).

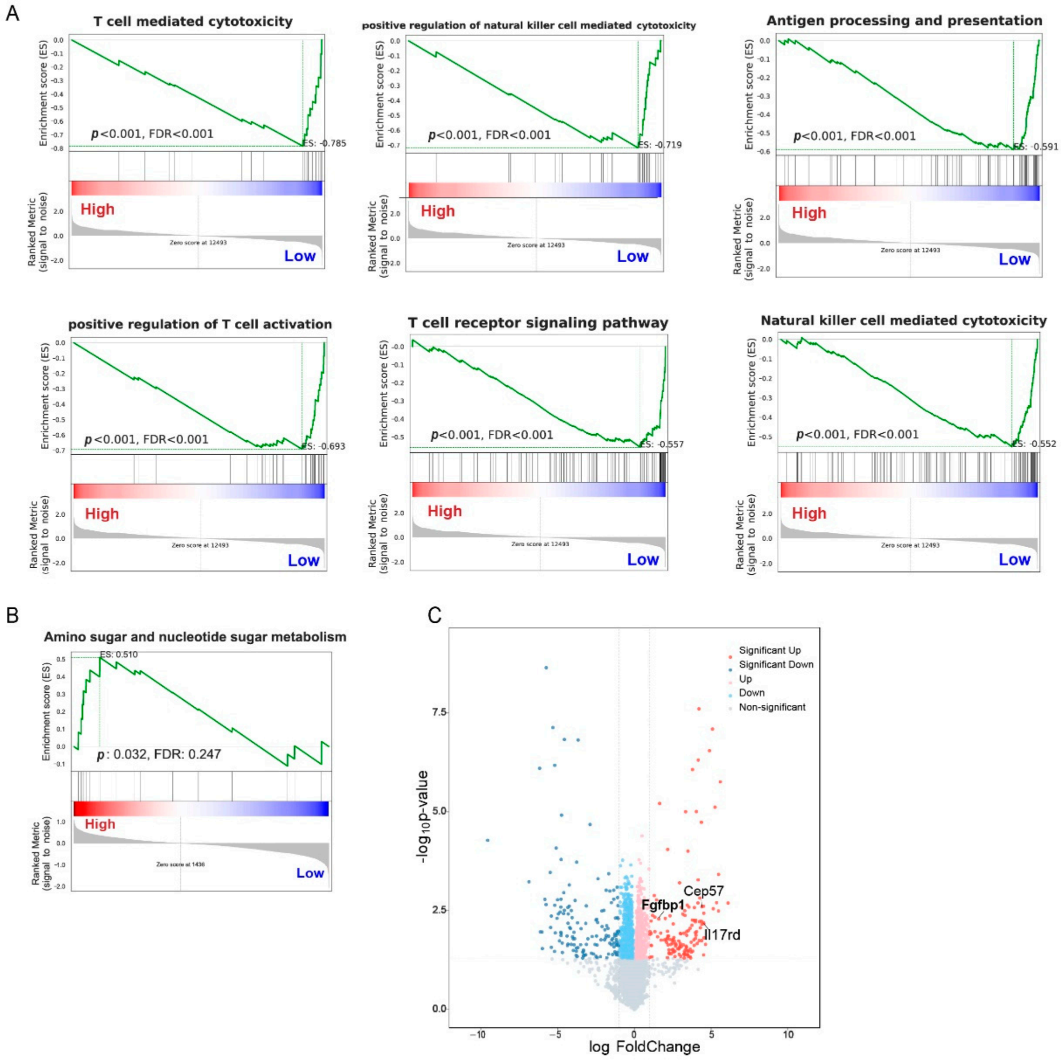

Supplementary Figure S2. Multi-omics results of tumors with high and low-proportion woundhealing CAFs. (A) GSEA analysis of some immune related pathways in high and low-proportion group. (B) GSEA analysis of amino sugar and nucleotide sugar metabolism in high and low-proportion group. (C) Volcano plot showed differences in protein expression between tumors with high and low-proportion woundhealing CAFs. FGFR related proteins including Cep57, Ll17rd, and Fgfbp1were marked.

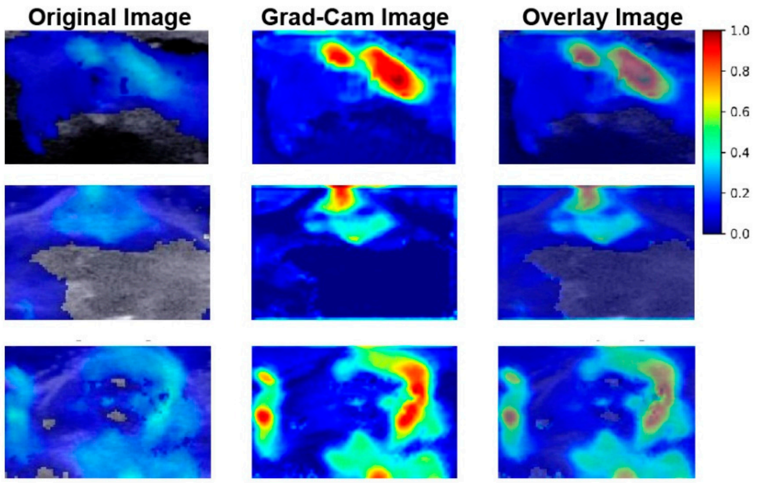

Supplementary Figure S3. Grad-CAM visualization results for several representative SWE images. From left to right, the original images, Grad-CAM images, and overlay images. The closer the color of a region is to red, the greater its contribution to the model's classification.

A

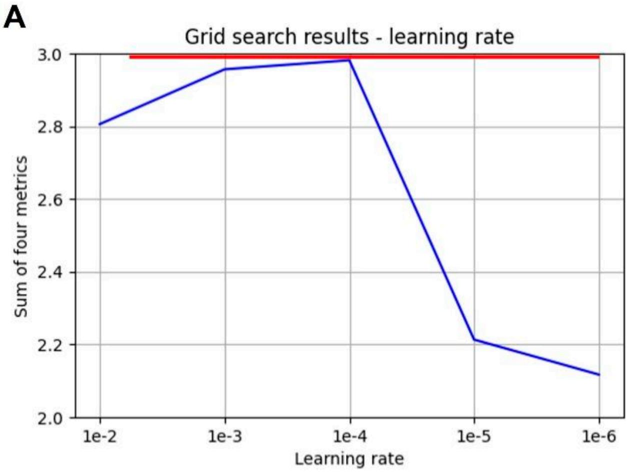

B

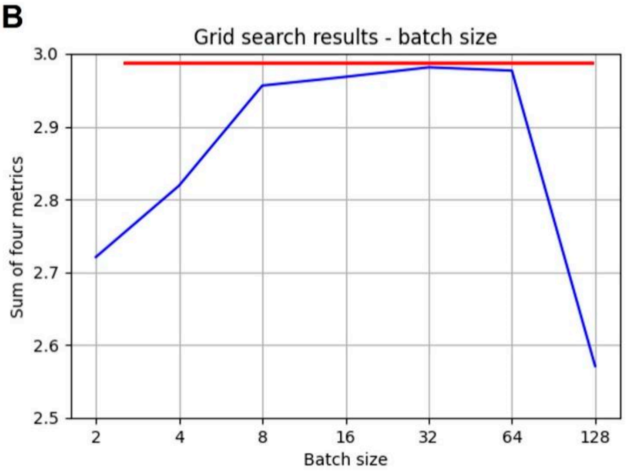

Supplementary Figure S4. The grid search results of hyperparameter tuning. (A) Hyperparameter tuning results of learning rate. (B) Hyperparameter tuning results of batch size.

	AUC	Accuracy	Sensitivity	Specificity
Image- level	0.8478±0.0113	0.7805±0.0244	0.8101±0.0390	0.7603±0.0576
Tumor-level	0.8509±0.0305	0.8285±0.0261	0.9200±0.0447	0.7455±0.0407

Supplementary Table S1. The cross validation results (mean±std) of our proposed deep learning model in WH CAF level prediction.
